# Oral Mucosa Derived α−Synuclein as a Potential Diagnostic Biomarker for Parkinson′s Disease

**DOI:** 10.3389/fnagi.2022.867528

**Published:** 2022-03-28

**Authors:** Yuanchu Zheng, Zhenwei Yu, Jiajia Zhao, Huihui Cai, Zhan Wang, Xuemei Wang, Tao Feng

**Affiliations:** ^1^Center for Movement Disorders, Department of Neurology, Beijing Tiantan Hospital, Capital Medical University, Beijing, China; ^2^Beijing Neurosurgical Institute, Capital Medical University, Beijing, China; ^3^China National Clinical Research Center for Neurological Diseases, Beijing, China

**Keywords:** Parkinson’s disease, α-synuclein, oral mucosa cells, biomarker, electrochemiluminescence, immunofluorescence

## Abstract

**Background:**

Pathological α-synuclein (α-Syn) is not only exclusive to the central nervous system (CNS) in Parkinson’s disease (PD), but also extended to biofluids and peripheral tissues including oral cavity. Both oral mucosa and nervous system are derived from ectodermal tissue, and potentially share common disease-specific characteristics. Oral mucosal exfoliative cytology is a non-invasive technique, which is an easily acceptable for patients and ordinary people. The purpose of this study was to determine the abnormal accumulation of α-Syn in oral mucosa of PD patients and to learn the diagnostic utility of oral mucosa α-Syn for PD.

**Methods:**

The oral mucosa samples were obtained from 57 patients with PD and 51 age-matched controls by cytological brush. Immunofluorescence analysis was used to investigate the presence and subcellular localization of α-Syn, phosphorylated α-Syn at Ser129 (pS129) and oligomeric α-Syn in the oral mucosa cells of PD patients and controls. Images taken as Z-stacks were analyzed for 3D reconstruction to visualize the α-Syn intracellular localization. Then, the concentrations of α-Syn, pS129, and oligomeric α-Syn in oral mucosa samples were measured using electrochemiluminescence assays.

**Results:**

Immunofluorescence images revealed the increased α-Syn, pS129, and oligomeric α-Syn levels in oral mucosa cells of PD patients than age-matched controls. The intracellular distributions of α-Syn species were determine by Z-stack images with 3D reconstruction, and α-Syn was detected in both the nucleus and cytoplasm. However, pS129 was mainly located in the cytoplasm, and oligomeric α-Syn was highly expressed in the nucleus and perinuclear cytoplasm. The concentrations of three α-Syn species were significantly increased in the oral mucosa cell samples of PD patients than controls (α-Syn, *p* = 0.001; pS129, *p* = 0.002; oligomeric α-Syn, *p* = 0.013). In PD patients, the oral mucosa α-Syn and oligomeric α-Syn levels were significantly correlated with Hoehn-Yahr scales (α-Syn, *r* = 0.495, *p* = 0.001; oligomeric α-Syn, *r* = 0.324, *p* = 0.03). The area under curve (AUC) of ROC analysis using an integrative model including α-Syn, pS129, and oligomeric α-Syn for PD diagnosis was 0.749, with 66.7% sensitivity and 72.5% specificity.

**Conclusion:**

This study for the first time demonstrated increased expressions of α-Syn, pS129, and oligomeric α-Syn in oral mucosa cells from PD patients, which serve as useful and non-invasive PD diagnostic biomarkers.

## Introduction

Parkinson’s disease is one of the most prevalent neurodegenerative diseases (Qi et al., 2021). The diagnosis of PD mostly relies on medical history and physical examination, which leads to a risk of misdiagnosis (Armstrong and Okun, 2020). The neuropathological features of PD are dopaminergic neurons loss in the substantia nigra pars compacta and abnormal deposition of intracellular α-Syn aggregations as Lewy bodies/neurites across the CNS (Poewe et al., 2017). Recent studies demonstrated that aggregated α-Syn is not only exclusive to the CNS, but also be found in biofluids and peripheral tissues such as blood, cerebrospinal fluid, olfactory mucosa, skin, salivary glands, retina, adrenal medulla, heart, and gastrointestinal tract (Ma et al., 2019b). Detection of pathological α-Syn in biofluids and peripheral tissues is a promising approach to PD diagnosis. However, most of the sampling methods are either invasive or of poor compliance. There is an urgent need for a non-invasive and easily acceptable sampling method for the biomarker development.

Our previous studies demonstrated that abnormal α-Syn deposition occurred in the periacinal space of minor salivary gland in PD patients (Gao et al., 2015; Ma et al., 2019a). We also found that the level of oligomeric α-Syn in salivary extracellular vesicles (EVs) is significantly higher in PD patients than healthy controls (Cao et al., 2019). Oral mucosa cells may share common disease-specific characteristics with neurons since it is derived from ectodermal tissue, which is also the embryonic origin of central nervous system (Paraskevaidi et al., 2020). A few studies have focused on Tau and β-amyloid in oral mucosa cells as biomarkers in neurological disorders such as AD and cognitive impairments (François et al., 2014, 2016; Leifert et al., 2015; Arredondo et al., 2017; Kose Ozlece et al., 2018; Zuev et al., 2019). These results suggested that the oral cavity, especially oral mucosa cells, may be a promising source of pathological α-Syn for PD. Oral mucosal exfoliative cytology is a simple and non-invasive technique to obtain oral mucosa cells (Paraskevaidi et al., 2020). In contrast to salivary glands, oral mucosal exfoliative cells sampling is safer and more acceptable. In addition, oral mucosal exfoliated cell samples contained more cellular content compared with saliva samples (Theda et al., 2018). Whether there is a differential α-Syn expression in oral mucosa cells between PD patients and age-matched controls deserves further study.

In this study, we intend to investigate the expression of α-Syn, pS129, and oligomeric α-Syn in oral mucosa cells from PD patients and age-matched controls, and to determine the diagnostic utility of α-Syn species in oral mucosa for PD. We also aim to identify the correlations between oral mucosa α-Syn levels and disease severity. To our knowledge, this is the first study to investigate the expression of α-Syn species in oral mucosa cells for PD.

## Materials and Methods

### Study Design and Subjects

Fifty-seven idiopathic PD patients and 51 age-matched controls were recruited from Beijing Tiantan Hospital, Capital Medical University from March 2021 to October 2021. All PD patients met the Movement Disorder Society Clinical Diagnostic Criteria for PD (Postuma et al., 2015). Exclusion criteria for PD group included diagnosis of atypical or secondary PD syndrome, severe head injury, a history of stroke, severe psychiatric disorders, severe systemic disorders, and oral mucosa diseases. Controls were excluded if they had a diagnosis of PD or other movement disorders, a family history of movement diseases, severe head injury, a history of stroke, severe psychiatric disorders, severe systemic disorders, and oral mucosa diseases. For all participants, a clinical and demographic data set were collected including age, sex, education, Mini-mental State Examination (MMSE), and Montreal Cognitive Assessment (MOCA). For PD patients, disease durations, Hoehn-Yahr staging scale, and Movement Disorder Society Unified Parkinson’s Disease Rating Scale Part III (MDS-UPDRS III) during OFF medication were used to assess the disease severity. The protocol of this cross-sectional study was reviewed and approved by the Ethics Committee of Beijing Tiantan Hospital, Capital Medical University. Informed consents were obtained from all participants.

### Oral Mucosa Sampling and Preparation

The participants were required to rinse their mouths with saline before sampling to avoid oral food residues and saliva contamination. Immediately after rinsing, two small-headed cytological brushes (2-cm head length) (Thomas et al., 2009) were used to collect oral mucosa samples from left and right inner check, respectively. Cytological brush sampling was performed in a circular expanding motion of brush head for 30 times. After oral mucosa sampling, thin smears were made on silane-coated microscope slides for immunofluorescence stains with one of the brushes. The slides were stored at −20°C. The other brush head was immersed in a 1.5 ml tube with 200 μl RIPA buffer (Applygen, cat. no. C1053+) for Electrochemiluminescence (ECL) immunoassays. The cytological brushes were removed after vortexing tubes for 1 min. The tubes were then sonicated for 1 min and clarified by centrifugation at 12000 × *g* and 4°C for 10 min. The oral mucosa cells protein-containing supernatant was transferred to a new tube and then stored at −80°C. Only at the time of analysis were the samples thawed.

The bicinchoninic acid (BCA) protein assay kit (Pierce/Thermo Fisher Scientific, Rockford, IL, United States) was used to assess protein concentrations in oral mucosa samples at an absorbance of 562 nm in comparison to a protein standard.

### Immunofluorescence Stains and Oral Mucosa Cells 3D Reconstruction

The slides were fixed in 4% paraformaldehyde for 10 min and then washed with Phosphate-buffered saline (PBS) for 3 times. After rinsing, slides were permeabilized in 1% Triton X-100 for 10 min. After 3 washings of 5 min each, slides were incubated in 5% Bovine serum albumin blocking solution (Sigma, Poole, United Kingdom). Recombinant anti-α-Syn MJFR-1 (ab138501, Abcam, Cambridge, MA, United States), recombinant anti-α-Syn aggregate MJFR-14 (ab209538, Abcam, Cambridge, MA, United States), and anti-phosphorylated α-Synuclein at Ser129 (825701, BioLegend, San Diego, CA, United States) antibodies were diluted 1:1000 in blocking solution and incubated overnight at 4°C. After washing with PBST buffer (PBS with 0.05% Tween^®^ 20) for 3 times, goat anti-mouse Alexa Fluor 488 (diluted 1:1000, ab150113, Abcam, Cambridge, MA, United States) and goat anti-rabbit Alexa Fluor 488 (diluted 1:1000, ab150077, Abcam, Cambridge, MA, United States) were incubated for 1 h at room temperature. Nuclei was stained with DAPI (0.2 μg/mL) for 5 min. Rhodamine Phalloidin (diluted 1:200, ca1610, Solarbio, Beijing, China) was used to display cytoskeletal range of oral mucosa cells. Slides observation was performed with a Zeiss LSM 700 confocal microscope (Carl Zeiss Microscopy GmbH, Oberkochen, Germany) using 40× objective. Images were taken as Z-stacks using 40× magnification for 3D reconstruction to visualize the localization of intracellular α-Syn.

### Electrochemiluminescence Immunoassays

Standard proteins, capture antibodies, and detection antibody used for all the α-Syn, pS129, and oligomeric α-Syn ECL immunoassays were described in our previous studies (Liu et al., 2019; Tian et al., 2019). Recombinant unphosphorylated a-Syn monomers (RP-001, Proteos, Inc., Kalamazoo, MI, United States), phosphorylated α-Syn monomers (RP-004, Proteos, Inc., Kalamazoo, MI, United States), and filaments (RP-002, Proteos, Inc., Kalamazoo, MI, United States) were used as standard proteins for all three a-Syn assays. Standard proteins were assessed by using NanoDrop OneC spectrophotometer (Thermo Scientific, Waltham, MA, United States) for concentration and diluted in Diluent 35 (D35, MSD, Rockville, MD, United States) to 1 μg/ml before preparation of the standard curve. Anti-α-Syn clone 42 (624096, BD Bioscience, San Jose, CA, United States) was labeled with Sulfo-TAG and used as detection antibodies for all a-Syn species. Recombinant anti-α-Syn MJFR-1 (ab 138501, Abcam, Cambridge, MA, United States), recombinant anti-α-Syn aggregate MJFR-14 (ab209538, Abcam, Cambridge, MA, United States), and anti-phosphorylated α-Synuclein at Ser129 (825701, BioLegend, San Diego, CA, United States) antibodies were biotinylated and used as capture antibodies. Capture antibodies were coated on Meso Scale Discovery (MSD, Rockville, MD, United States) U-Plex plates by incubating the plates with capture antibody solutions for 1 h with 600 rpm shaking at room temperature. After rinsing three times with 150 μl wash buffer (MSD, Rockville, MD, United States), plates were blocked with 150 μl D35 for 1 h with 600 rpm shaking at room temperature, then rinsed three times again. The oral mucosa cells protein-containing samples were diluted 1:2.5 in D35 and incubated together with standard proteins for 1 h while shaking at 600 rpm. After rinsing three times, detection antibody solution (1 μg/ml) was loaded and incubated for 1 h with 600 rpm shaking. Immediately after rinsing with wash buffer for three times, 150 μl 2 × Reading Buffer (MSD, Rockville, MD, United States) was added and the plates were analyzed in a Sector Imager 6000 (MSD, Rockville, MD, United States).

### Statistical Analysis

Statistical analysis was performed using SPSS 22.0 software (IBM, Chicago, IL, United States) and GraphPad Prism 8 (GraphPad Software, La Jolla, CA, United States). Before analysis, concentrations of α-Syn, pS129, and oligomeric α-Syn was normalized to total oral mucosa cells protein levels. Non-parametric Mann Whitney *U* test was used to compare group means and Spearman’s rank correlation coefficient was used to analyze correlation between biomarkers and disease severity. *P* < 0.05 was considered significant. Binary logistic regression was used to create a multivariable logistic regression model suited for analyzing independent influencing factors for PD diagnosis. The area under the receiver operating characteristic (ROC) curve was analyzed and Youden index maxima (sensitivity + specificity − 1) was calculated to obtain the most optimum cutoff values for PD and controls.

## Results

### Demographic and Clinical Features

A total of 57 PD patients and 51 age-matched controls were recruited in this study. The demographic and clinical data of all subjects were listed in Table 1. There was no difference in mean sex, age, MMSE, MOCA distribution between PD patients and controls. The median disease duration of PD patients was 5 years (range 7 months–16 years), the median Hoehn-Yahr scale during OFF medication was 3 (range 1–5) and median MDS-UPDRS III score during OFF medication was 41.5 (range 5–74).

**TABLE 1:** Demographic and clinical data.

Group	PD, *n* = 57	HC, *n* = 51	*p*
Females/Males	25/32	27/24	0.346
Age (year)	62.11 ± 10.30	62.94 ± 6.35	0.732
Duration (year)	6.18 ± 3.69	NA	NA
Hoehn-Yahr	2.90 ± 0.80	NA	NA
MDS-UPDRS III	42.50 ± 16.00	NA	NA
MMSE	26.91 ± 3.32	28.06 ± 1.77	0.163
MOCA	23.55 ± 4.73	24.84 ± 2.34	0.394

*PD, Parkinson’s Disease; HC, Healthy Controls; MMSE, Mini-mental State Examination; MOCA, Montreal Cognitive Assessment; MDS-UPDRS III, Movement Disorder Society Unified Parkinson’s Disease Rating Scale Part III. Data are represented as mean ± SD.*

### Expression of α-Syn Species in Oral Mucosa Cells Between Parkinson’s Disease and Controls

The expression of α-Syn in the oral mucosa samples was determined by MJFR1 (ab138501, Abcam, Cambridge, MA, United States), which is targeting a recombinant full-length human α-Syn protein. PS129 (cat825701, BioLegend, San Diego, CA, United States) antibody is against a synthetic peptide corresponding to amino acids 124–134 of α-Syn, phosphorylated at Serine 129. The expression of oligomeric α-Syn were determined by MJFR-14-6-4-2 (ab209538, Abcam, Cambridge, MA, United States), which is conformation specific to the α-Syn aggregates.

**FIGURE 1:**
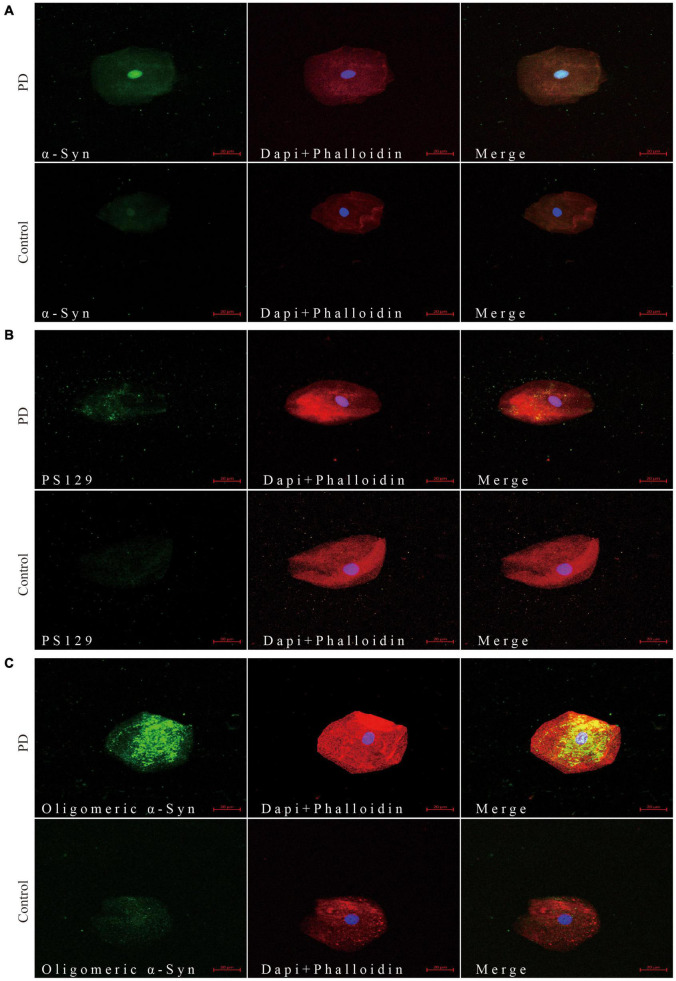
Confocal microscopy (×40) study of α-Syn **(A)**, pS129 **(B)**, and oligomeric α-Syn **(C)** expression pattern in oral mucosa cells of PD patients and controls. The first column shows α-Syn **(A)**, pS129 **(B)**, and oligomeric α-Syn **(C)** distribution in green. The cytoskeleton stained with phalloidin (red) and nuclear stained with DAPI (blue) are shown in second column. Merge images are shown in third column. Scale bar: 20 μm. PD, Parkinson’s Disease; α-Syn, α-synuclein; pS129, phosphorylated α-Syn at Ser129.

Through confocal microscopy analysis, α-Syn (Figure 1A), pS129 α-Syn (Figure 1B), and oligomeric α-Syn (Figure 1C) immunoreactive signals were detectable in oral mucosa cells of PD patients and controls. Immunofluorescence imaging revealed significantly increased immunoreactive signals of α-Syn, pS129, and oligomeric α-Syn in oral mucosa cells of PD patients than those in controls. Alpha-Syn (MJFR1) showed a diffuse distribution. PS129 showed a dotted positivity. Oligomeric α-Syn showed a predominantly granular positivity.

**FIGURE 2:**
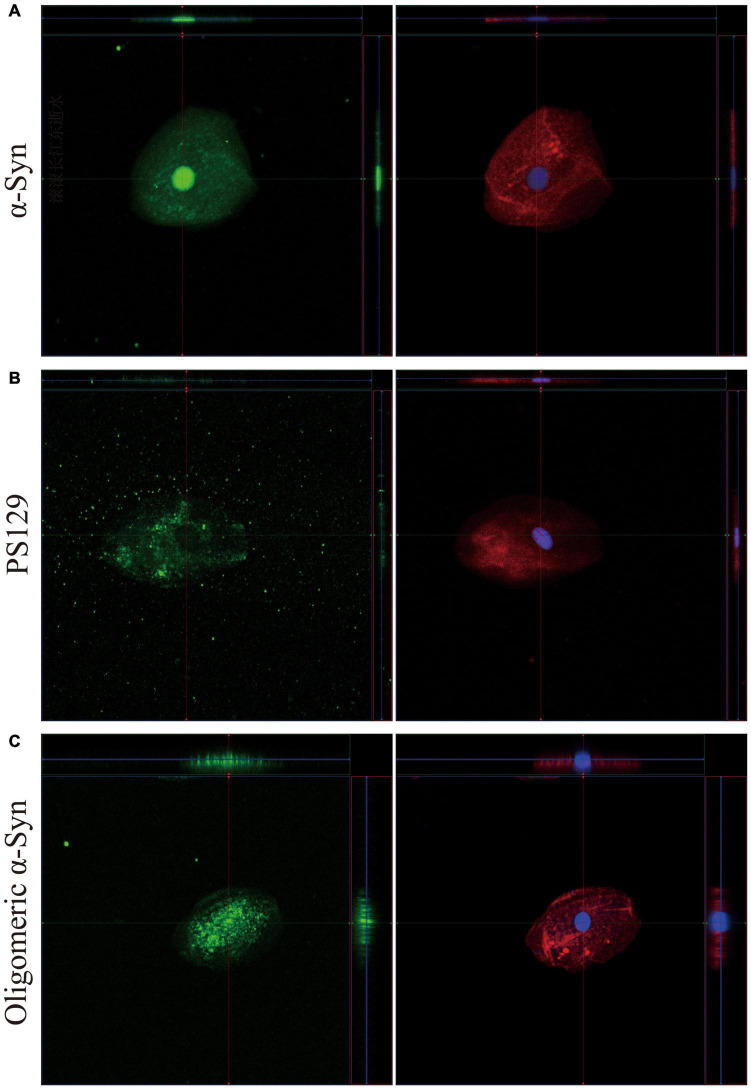
3D reconstruction of z-stack confocal images (×40). The first column shows α-Syn **(A)**, pS129 **(B)**, and oligomeric α-Syn **(C)** in green. The cytoskeleton stained with phalloidin (red) and nuclear stained with DAPI (blue) are shown in second column. α-Syn, α-synuclein; pS129, phosphorylated at Ser129.

3D reconstruction of Z-stack confocal images revealed different intracellular distributions among α-Syn, pS129, and oligomeric α-Syn in oral mucosa cells. But there was no difference in α-Syn intracellular distribution between PD patients and controls. More specifically, the expression pattern of α-Syn was mainly detected in the nucleus and cytoplasm of oral mucosa cells (Figure 2A). PS129 was mainly located in the cytoplasm of oral mucosa cells (Figure 2B). Oligomeric α-Syn was highly expressed in the nucleus and perinuclear cytoplasm of oral mucosa cells (Figure 2C).

### The Concentrations of α-Syn in Oral Mucosa Cells and Correlations With Disease Severity

**FIGURE 3:**
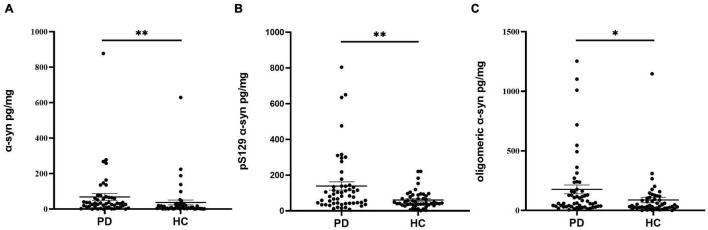
The concentrations of α-Syn, pS129, and oligomeric α-Syn in oral mucosa samples of PD patients and controls. **(A)** α-Syn, normalized to total oral mucosa cells proteins (pg/mg); ^**^p = 0.001 (Mann Whitney *U* test); **(B)** pS129, normalized to total oral mucosa cells proteins (pg/mg); ^**^p = 0.002 (Mann Whitney *U* test); **(C)** oligomeric α-Syn, normalized to total oral mucosa cells proteins (pg/mg); *p = 0.013 (Mann Whitney *U* test).

The concentrations of α-Syn, pS129, and oligomeric α-Syn were normalized to total oral mucosa cells protein levels before analysis. Compared with controls, the concentration of α-Syn in oral mucosa cells was significantly higher in PD patients (*p* = 0.001, Figure 3A and Table 2). The pS129 and oligomeric α-Syn levels were also increased in PD group compared with those in controls (*p* = 0.002, *p* = 0.013, Figures 3B,C and Table 2). In PD group, both the α-Syn and oligomeric α-Syn levels in oral mucosa cells significantly correlated with Hoehn-Yahr scales (Correlation Coefficient *r* = 0.495, *r* = 0.324; *p* = 0.001, *p* = 0.03). However, pS129 levels did not correlated with Hoehn-Yahr scales. The α-Syn, pS129, and oligomeric α-Syn levels did not correlate with disease durations or MDS-UPDRS III scores.

**TABLE 2:** The concentrations of α-Syn in oral mucosa cells.

Group	PD	HC	*p*
α-Syn, pg/mg	30.13 [11.29–64.20]	9.60 [2.73–24.35]	0.001*
pS129 α-Syn, pg/mg	82.06 [42.13–138.21]	43.06 [33.37–84.59]	0.002*
Oligomeric α-Syn, pg/mg	63.31 [31.75–166.32]	38.04 [20.09–104.08]	0.013*

PD, Parkinson’s Disease; HC, Healthy Controls; α-Syn, α-synuclein; pS129, phosphorylated α-syn at Ser129. Data are represented as median [25–75%]. *This p-value indicates a statistically significant difference.

### Receiver Operating Characteristic Curve Analysis of α-Syn Species in Oral Mucosa Cells Between Parkinson’s Disease Patients and Age-Matched Controls

**FIGURE 4:**
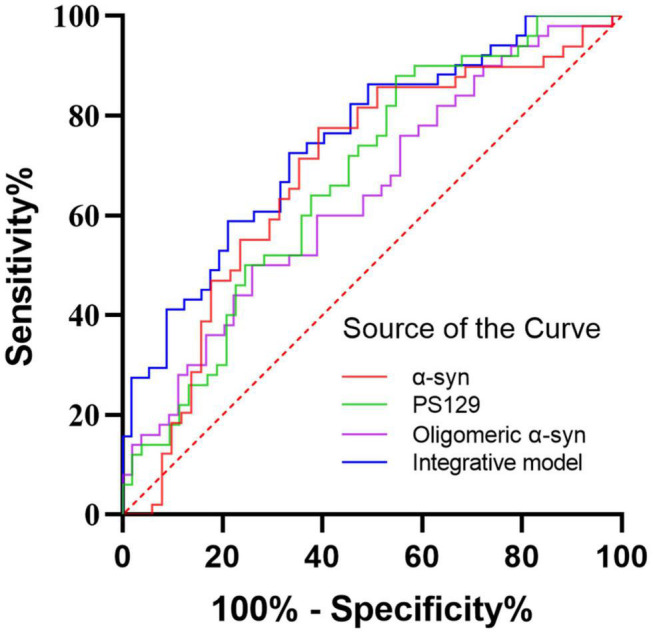
The ROC curves for α-Syn, pS129, oligomeric α-Syn, and the integrative model. Red curve: α-Syn (AUC = 0.684); Green curve: pS129 α-Syn (AUC = 0.674); Purple curve: oligomeric α-Syn (AUC = 0.641); Blue curve: the integrative model including α-Syn, pS129 α-Syn, and oligomeric α-Syn (AUC = 0.749).

Receiver operating characteristic analysis was performed to assessed the diagnostic performance of α-Syn species in oral mucosa cells for PD (Figure 4). Alpha-Syn discriminated PD from controls with an AUC of 0.684 and sensitivity of 60.8% and a specificity of 77.6%. The AUC of pS129 discriminating PD from controls was 0.674, with a sensitivity of 45.3% and a specificity of 88.0%. The AUC of oligomeric α-Syn discriminating PD from controls was 0.641, with a sensitivity of 74.1% and a specificity of 50.0%. The binary logistic regression with backward like ratio was used to create a multivariable logistic regression model based on the levels of α-Syn, pS129, and oligomeric α-Syn in oral mucosa samples. The AUC of this integrative model was 0.749, with 66.7% sensitivity and 72.5% specificity.

## Discussion

In the present study, we demonstrated the differential expression of α-Syn, pS129, and oligomeric α-Syn in oral mucosa cells between PD patients and controls. Immunofluorescence imaging revealed greater α-Syn accumulation in oral mucosa cells of PD patients than those in controls. The further ECL assays validated the immunofluorescence imaging results. The levels of α-Syn, pS129, and oligomeric α-Syn were significantly increased in oral mucosa samples of PD patients compared with those in controls. Taken together, these data suggested that the expression pattern of α-Syn in oral mucosa may be a potential biomarker for PD.

Immunofluorescence imaging revealed the abnormal accumulation of α-Syn, pS129 α-Syn, and oligomeric α-Syn in oral mucosa cells of PD patients and relatively faint positivity in control group. Alpha-Syn is a neuronal protein that regulates synaptic vesicle trafficking and subsequent neurotransmitter release (Serratos et al., 2021). It is abundant in the brain, but also be found in the skin, heart, salivary glands, retina, gastrointestinal tract, and olfactory mucosa (Ma et al., 2019b). In brain, α-Syn is mainly found in neural cells of the neocortex, hippocampus, substantia nigra, thalamus, and cerebellum, but it was also found in glia cells (Braak et al., 2003; Koga et al., 2021). Oral mucosa cells may share common α-Syn expression pattern with neurons since it is derived from ectodermal tissue which is also the embryonic origin of CNS (Paraskevaidi et al., 2020). Consistent with the distribution pattern in brain, α-Syn in peripheral tissues was mainly located in nerve fibers of peripheral tissues (Donadio et al., 2014; Gelpi et al., 2014). But several studies reported the expression of α-Syn in non-neural mucosa cells, such as olfactory mucosa and enteric mucosa (Brozzetti et al., 2020; Casini et al., 2021). In a recent *in vivo* study, Brozzetti et al. (2020) demonstrated the presence of α-Syn and several other neurodegeneration-associated proteins in both neuronal and non-neuronal olfactory mucosa cells of healthy individuals. Brozzetti et al. used a monoclonal antibody generated from the non-modified full length of α-Syn, which is similar to the anti-α-Syn antibody (ab 138501, Abcam, Cambridge, MA, United States) used in the present study. In addition, oral mucosa shares the common embryonic origin with olfactory mucosa, which may have the similar expression pattern of neurodegeneration-associated proteins. In another study, Casini et al. found that α-Syn was not only present in the enteric nerves, but also be found in the epithelial mucosal cells of normal human jejunum samples (Casini et al., 2021). These data supported the expression of α-Syn in non-neural mucosa cells. As for the pathological α-Syn deposition, pS129, and oligomeric α-Syn were detectable in controls’ oral mucosa cells with a relatively faint positivity in the present study. In several previous studies, researchers also reported the pathological α-Syn deposition in normal controls’ peripheral tissues. Barrenschee et al. (2017) found that pS129 immunoreactive signals were detectable within the submucosal plexus in both PD and controls. Wang et al. (2020) also reported faint but detectable pS129 immunoreactive signals in controls’ skin nerve. The anti-pS129 antibodies used in the previous and present studies were both directed against phosphorylated α-Syn. Mazzetti et al. reported a significant increase in skin oligomeric α-Syn expression in consecutive PD patients compared to consecutive healthy controls. Although ∼1/3 of the consecutive healthy controls had no skin oligomeric α-Syn expression at all, there were still a large proportion of consecutive healthy controls with faint skin oligomeric α-Syn expression (Mazzetti et al., 2020). Recently, the measurement of oligomeric α-Syn in biofluid has emerged as a promising approach to diagnose PD. Several studies have reported higher oligomeric α-Syn levels or ratio of oligomeric/total α-Syn in cerebrospinal fluid and red blood cells in PD patients than in healthy controls (Tian et al., 2019; Majbour et al., 2021). Notably, oligomeric α-Syn were also detected in biofluids from healthy control individuals although at lower concentrations. These results suggested that pathological α-Syn deposition was detectable in peripheral tissues and biofluids of normal controls with relatively faint positivity.

Differential expression between PD and controls made oral mucosa α-Syn a promising biomarker for PD. Immunofluorescence imaging revealed the increased immunopositivity of α-Syn, pS129, and oligomeric α-Syn in oral mucosa cells of PD patients than those in controls. To further quantify the α-Syn levels in oral mucosa cells, a highly sensitive ECL immunoassay was used in the present study. ECL immunoassay is much more sensitive and accurate than traditional ELISA, with the detection range down to 5–10 pg/ml (Tian et al., 2019). It is based on the binding of specific antibodies to the analyte within electron-enriched wells, with the subsequent generation of an electrochemiluminescent signal (Parnetti et al., 2019). This assay was a home-brewed kit carried out by our collaborators to qualify α-Syn, pS129, and oligomeric α-Syn (Tian et al., 2019). Consistent with immunofluorescence images, the levels of α-Syn, pS129, and oligomeric α-Syn were significantly increased in oral mucosa cells of PD patients compared with those in controls. Both immunofluorescence and ECL immunoassay demonstrated the existence of abnormal α-Syn accumulations in oral mucosa cells from PD patients.

One possible explanation for the abnormal α-Syn accumulation in oral mucosa cells is the changed oral environment. In several recent studies, saliva and subgingival dental plaque microbiome in PD patients was found to be altered from those in controls (Fleury et al., 2021; Rozas et al., 2021). Dysphagia, drooling, and salivary pH were factors that significantly influenced oral microbiota (Rozas et al., 2021). Also, the levels of IL-1β and IL-1RA were significantly increased in the gingival crevicular (Fleury et al., 2021). It suggested that alterations in oral microbiota in PD patients may trigger the misfolding of α-syn through inflammatory response, which eventually leads to the abnormal accumulation of α-Syn.

A meta-analysis of *in vivo* α-Syn deposits as diagnostic biomarkers for PD has demonstrated that skin biopsy examination using anti-phosphorylated α-Syn antibody has the highest pooled sensitivity of 0.76 and pooled specificity of 1.00 over other specimens (Tsukita et al., 2019). In a recent study, Donadio et al. (2021) also found that skin biopsy immunofluorescence presented higher diagnostic accuracy than skin real-time quaking-induced conversion. Based on the quantitative results of α-Syn, pS129, and oligomeric α-Syn in oral mucosa samples, we developed a multivariate PD diagnostic model. The AUC of this multivariate model is 0.749, with 66.7% sensitivity and 72.5% specificity. Though the overall sensitivity was moderate, utilization of α-Syn in oral mucosa as a biomarker for PD have several advantages over other specimens. Firstly, the collection process of oral mucosa is non-invasive, which is easily acceptable to patients and ordinary people. Compared with saliva, oral mucosa cells allow visualization of intracellular expression of neurodegeneration associated proteins and contain more cellular content (Theda et al., 2018). Moreover, as the oral mucosa cells turn over every 7–21 days, repeated samplings in a short period of time are easy to achieve.

There was no difference in the intracellular distributions of α-Syn species between PD and controls. 3D reconstruction of z-stack confocal images revealed different intracellular distributions among α-Syn, pS129, and oligomeric α-Syn in oral mucosa cells. More specifically, the expression pattern of α-Syn was mainly detected in the nucleus and cytoplasm of oral mucosa cells, oligomeric α-Syn was highly expressed in the nucleus and perinuclear cytoplasm of oral mucosa cells, pS129 was mainly located in the cytoplasm of oral mucosa cells. Alpha-Syn was named because of its localization to the nucleus and presynaptic terminals of neurons (Maroteaux et al., 1988). Since then, a number of additional intracellular localizations for α-Syn have been reported, including mitochondria, endoplasmic reticulum, golgi body, and cytoskeleton (Burré et al., 2018). Alpha-Syn shows the characteristic of widespread intraneural distribution. As for the intracellular distribution of α-Syn in mucosa cells, a previous study reported the localization to cellular membrane in olfactory mucosa (Brozzetti et al., 2020). This is inconsistent with our findings in the present study that α-Syn was mainly detected in the nucleus and cytoplasm of oral mucosa cells. The inconsistency may be due to different primary antibody types or mucosa cell types. The intracellular expression patterns of α-Syn in mucosa cells need to be further studied.

There are several limitations to this study. Firstly, we did not recruit multiple system atrophy (MSA) patients in this study. As another common α-synucleinopathy, the α-Syn expression pattern in oral mucosa cells of MSA patients deserves further study. We are now recruiting MSA patients for the further study. Secondly, most of the PD patients included in this study were in the disease middle and late stages. Therefore, it is impossible to determine how early the deposition of α-Syn in oral mucosa cells occurs in PD. Further longitudinal cohort studies are needed to determine whether α-Syn in oral mucosa cells is a potential progression biomarker for PD.

In conclusion, the present study demonstrated the presence of α-Syn, pS129, and oligomeric α-Syn in oral mucosa cells of both PD patients and controls. PD patients had greater α-Syn accumulation compared with controls in oral mucosa cells. The concentrations of α-Syn, pS129, and oligomeric α-Syn increased in PD oral mucosa cells and yield the first evidence for the potential of oral mucosa α-Syn species as diagnostic biomarkers of PD. Alpha-Syn expression pattern in oral mucosa cells of prodromal PD patients and other α-synucleinopathies such as MSA and dementia with Lewy bodies deserve further studies.

## Data Availability Statement

The original contributions presented in the study are included in the article/supplementary material, further inquiries can be directed to the corresponding author.

## Ethics Statement

The studies involving human participants were reviewed and approved by Ethics Committee of Beijing Tiantan Hospital, Capital Medical University, Beijing, China. The patients/participants provided their written informed consent to participate in this study.

## Author Contributions

TF and YZ designed the study. ZY and YZ performed the measurements, data analysis, and wrote the manuscript. JZ, HC, ZW, and XW contributed to sample collection and preparation. All authors contributed to the article and approved the submitted version.

## Conflict of Interest

The authors declare that the research was conducted in the absence of any commercial or financial relationships that could be construed as a potential conflict of interest.

## Publisher’s Note

All claims expressed in this article are solely those of the authors and do not necessarily represent those of their affiliated organizations, or those of the publisher, the editors and the reviewers. Any product that may be evaluated in this article, or claim that may be made by its manufacturer, is not guaranteed or endorsed by the publisher.
